# Supplementary material for: Exceptional longevity of mammalian ovarian and oocyte macromolecules throughout the reproductive lifespan
Source: bioRxiv. 2023 Oct 20:2023.10.18.562852. Preprint. [Version 1] doi: 10.1101/2023.10.18.562852 (PMC10614913; doi:10.1101/2023.10.18.562852)
Supplement: Supplement 1 [file NIHPP2023.10.18.562852v1-supplement-1.pdf]

bioRxiv preprint doi: https://doi.org/10.1101/2023.10.18.562852; this version posted October 20, 2023. The copyright holder for this preprint (which was not certified by peer review) is the author/funder, who has granted bioRxiv a license to display the preprint in perpetuity. It is made available under aCC-BY 4.0 International license.

Supplementary Figure 1

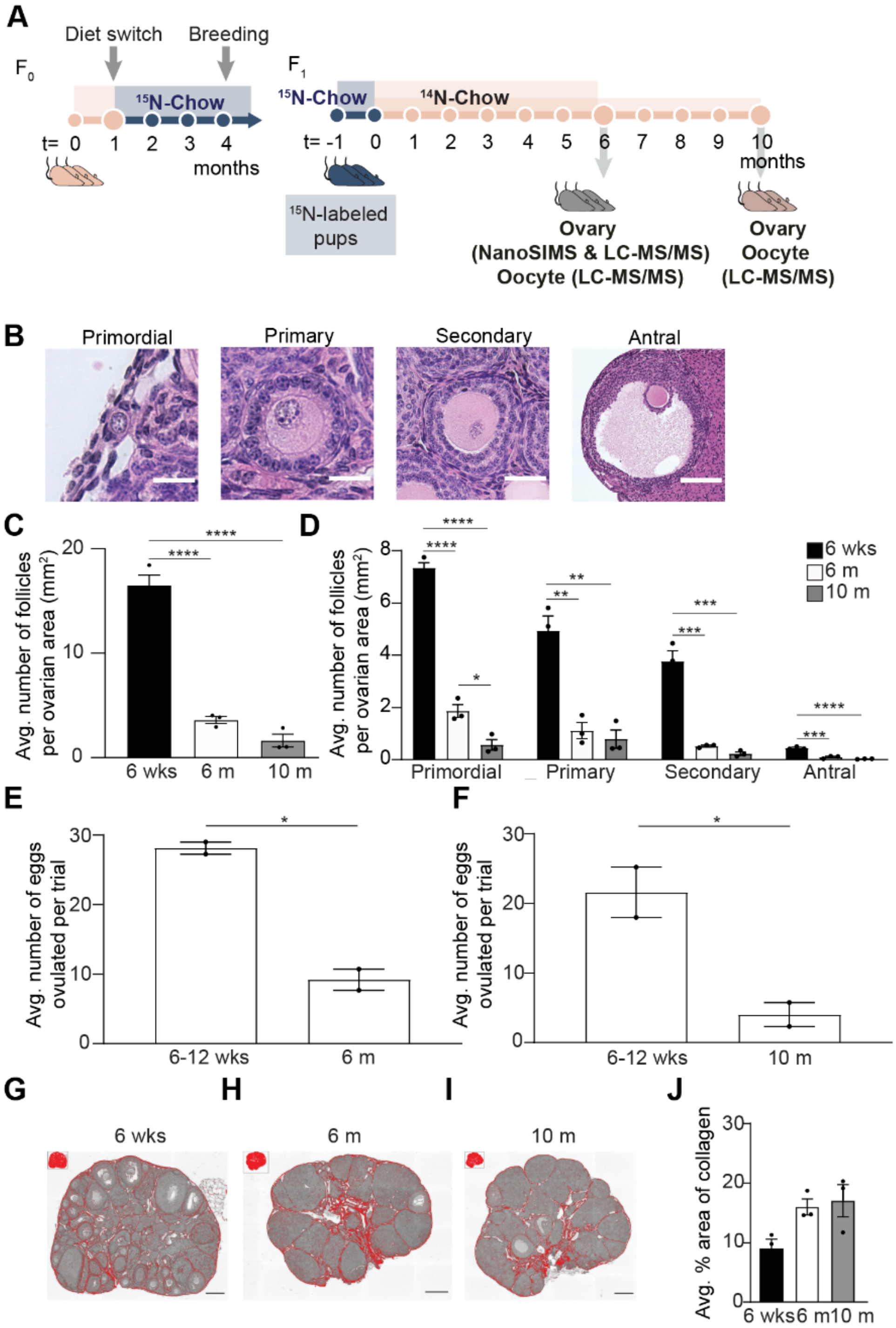

<u>Fig. S1:</u> Multi-generational whole animal pulse-chase labeling design along the reproductive aging continuum. (A) Wild-type female FVB mice (n=3) were fed a ^{15}N-diet for 13 weeks and were maintained on a ^{15}N-labeled diet through breeding, pregnancy, and weaning to produce ^{15}N-labeled pups. Labeled

bioRxiv preprint doi: https://doi.org/10.1101/2023.10.18.562852; this version posted October 20, 2023. The copyright holder for this preprint (which was not certified by peer review) is the author/funder, who has granted bioRxiv a license to display the preprint in perpetuity. It is made available under aCC-BY 4.0 International license.

pups were sacrificed after switching over to a ^{14}N-diet for a chase period of 6- or 10-months. Ovaries and oocytes were designated for NanoSIMS, or liquid-chromatography/mass spectrometry. Female FVB mice experience age-associated changes in ovarian reserve, ovarian microenvironment, and gamete quality. **(B)** Representative images of each follicle class from mice of 6 weeks old and 6 months old. **(C)** Average follicle number per area of ovarian section from mice of the following ages: 6 weeks, 6 months, and 10 months (N= 3 mice per age cohort). **(D)** Graph represents average number of follicles within each follicle class per area of ovarian section for mice ages 6 weeks, 6 months, and 10 months. (E) Comparison of average number of eggs ovulated per trial for mice at 6-12 wks, 6 months, and (F) 10 months. Representative processed color threshold images of PSR-stained ovarian tissue sections from mice **(G)** 6 weeks old, **(H)** 6 months old, and **(I)** 10 months old**. (J)** Graph comparing the average percent area of PSR-positive staining per ovarian section (pixels/μm^2). Data are shown as mean±SEM. Statistical analysis was performed using a one-way ANOVA. Asterisk denotes statistical significance (* $p \leq .05$; ** $p \leq .01$; *** $p \leq .001$; **** $p \leq .0001$). Scale bar for images of (B) primordial, primary, secondary, and antral follicles are 20 μm, 40 μm, and 140 μm, respectively. Scale bars in (G-I) are 180 μm.

bioRxiv preprint doi: https://doi.org/10.1101/2023.10.18.562852; this version posted October 20, 2023. The copyright holder for this preprint (which was not certified by peer review) is the author/funder, who has granted bioRxiv a license to display the preprint in perpetuity. It is made available under aCC-BY 4.0 International license.

Supplementary Figure 2

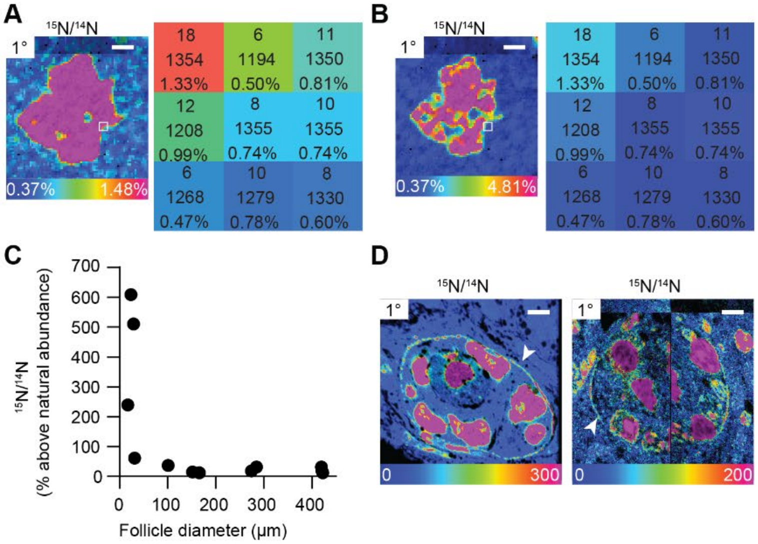

Fig. S2: Multi-isotope imaging mass spectrometry (MIMs) uncovers structures enriched with ^{15}N. (A) Hue saturation intensity image maps ^{15}N/^{14}N ratio across nuclear region of a primary follicle. Using a rainbow scale, blue is set to the natural ratio of ^{15}N (0.37%) and overabundance is set to 1.48% (or 300% above the natural ratio). Each pixel provides quantitative information. The numbers in each pixel represent the number of ^{15}N ions, ^{14}N ions, and the ^{15}N/^{14}N ratio, respectively. **(B)** Changes to the rainbow scale can be used to emphasize regional ratio differences and change the visual representation of the data. Visual changes to HSI images due to different scales, does not change the quantitative data behind each pixel. **(C)** Total ^{15}N abundance of each follicle plotted by follicle diameter shows smaller follicles containing high abundance of ^{15}N. **(D)** HSI images reveal high ^{15}N abundance concentrated at the basement membrane of early-stage follicles. Scale bar (panel A-B) =2.5 µm, (D) left= 4.0 µm, (D) right = 4.5 µm.

bioRxiv preprint doi: https://doi.org/10.1101/2023.10.18.562852; this version posted October 20, 2023. The copyright holder for this preprint (which was not certified by peer review) is the author/funder, who has granted bioRxiv a license to display the preprint in perpetuity. It is made available under aCC-BY 4.0 International license.

Supplementary Figure 3

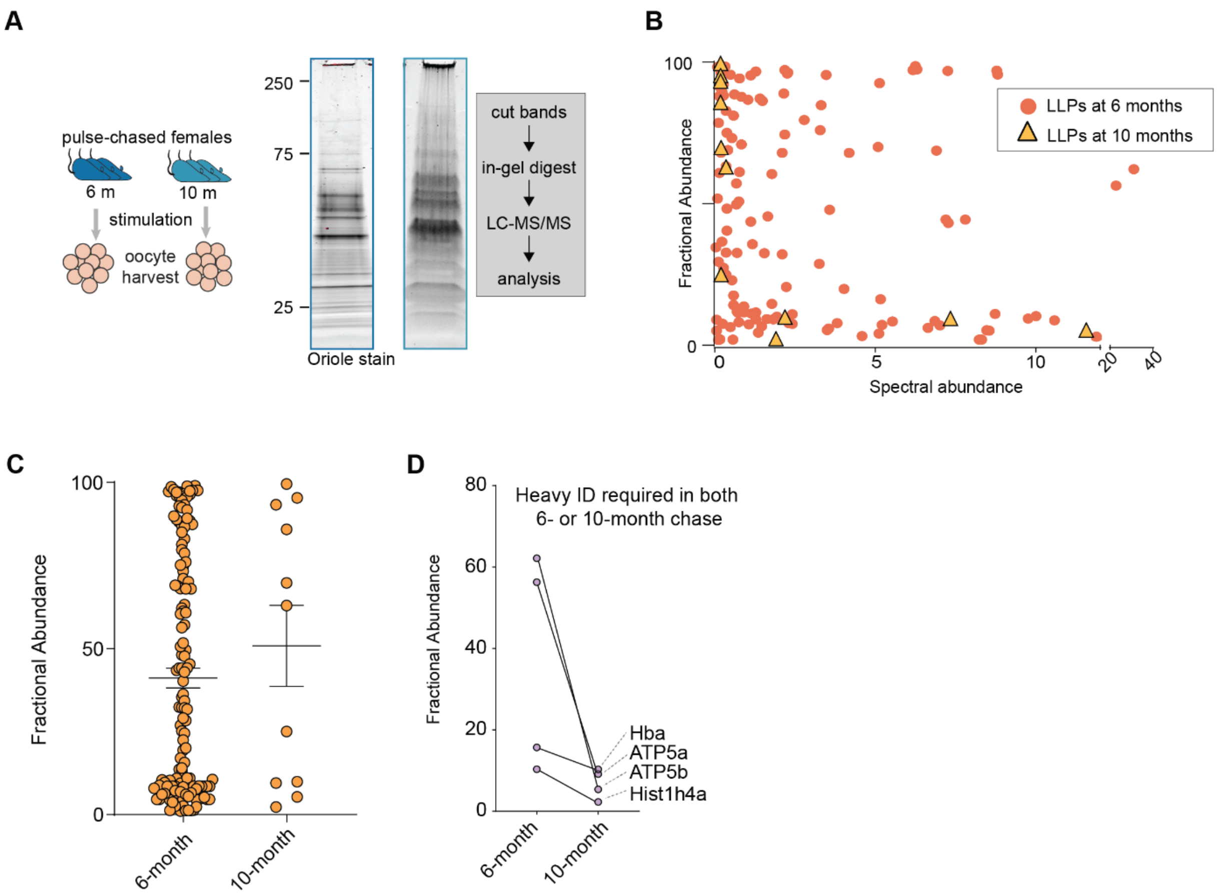

384
385 **<u>Fig. S3:</u> Long-lived proteins at 6- and 10-months chase points in oocytes. (A)** Experimental scheme to
386 identify and measure LLPs mouse oocyte. **(B-C)** Fractional abundance of each identified LLPs at both 6
387 months and 10 months as compares to the spectral abundance for each LLPs. **(D)** Plots showing decrease
388 in FA values for same proteins identified as LLPs at 6 and 10-month chase.
